# Long-Term Impacts of Diurnal Temperature Range on Mortality and Cardiovascular Disease: A Nationwide Prospective Cohort Study

**DOI:** 10.3390/metabo12121287

**Published:** 2022-12-19

**Authors:** Haosu Tang, Xin Wang, Yuting Kang, Congyi Zheng, Xue Cao, Yixin Tian, Zhen Hu, Linfeng Zhang, Zuo Chen, Yuxin Song, Runqing Gu, Jiayin Cai, Gang Huang, Zengwu Wang

**Affiliations:** 1 State Key Laboratory of Numerical Modeling for Atmospheric Sciences and Geophysical Fluid Dynamics (LASG), Institute of Atmospheric Physics, Chinese Academy of Sciences, Beijing 100045, China; 2 Laboratory for Regional Oceanography and Numerical Modeling, Qingdao National Laboratory for Marine Science and Technology, Qingdao 266005, China; 3 University of Chinese Academy of Sciences, Beijing 100045, China; 4 Division of Prevention and Community Health, National Center for Cardiovascular Disease, National Clinical Research Center of Cardiovascular Disease, State Key Laboratory of Cardiovascular Disease, Fuwai Hospital, Peking Union Medical College, Chinese Academy of Medical Sciences, Beijing 100006, China; 5 Office of the National Clinical Research Center for Geriatric Diseases, Beijing Hospital, National Center of Gerontology, Institute of Geriatric Medicine, Chinese Academy of Medical Sciences, Beijing 100006, China

**Keywords:** diurnal temperature range, mortality, cardiovascular disease, cohort study, future projection

## Abstract

Previous studies have documented the associations between short-term diurnal temperature range (DTR) exposure and cardiovascular disease (CVD) via time-series analyses. However, the long-term impacts of DTR through a population-based prospective cohort have not been elucidated thoroughly. This study aimed to quantify the longitudinal association of DTR exposure with all-cause mortality and CVD in a nationwide prospective cohort and, by extension, project future DTR changes across China under climate change. We included 22,702 adults (median age 56.1 years, 53.7% women) free of CVD at baseline from a nationwide cross-sectional study in China during 2012–2015, and examined three health outcomes during a follow-up survey in 2018–2019. We estimated the chronic DTR exposure as baseline annual mean daily maximum minus minimum temperature. The Cox proportional hazards regression was adopted to assess the multivariable-adjusted hazard ratio and its corresponding 95% confidence interval (95% CI). We employed 31 downscaled global climate models under two shared socioeconomic pathways for future projection. During the median follow-up period of ~5 years, 1096 subjects died due to all causes while 993 and 597 individuals developed fatal or nonfatal CVD and fatal or nonfatal stroke, respectively. The cumulative incidence rates of all-cause mortality, CVD, and stroke were 10.49, 9.45, and 5.64 per 1000 person-years, respectively. In the fully adjusted models, the risks for all-cause mortality, CVD, and stroke would increase by 13% (95% CI: 8–18%), 12% (95% CI: 7–18%), and 9% (95% CI: 2–16%) per 1 °C increment in DTR, respectively. Moreover, linear positive associations for the concentration–response curves between DTR and mortality and CVD were observed. We also found significantly greater DTR-related mortality risks among rural residents than their urban counterparts. The DTR changes featured a dipole pattern across China under a warming climate. The southern (northern) China would experience increased (decreased) DTR exposure by the end of 21st century. The present study indicates that chronic DTR exposure can exert long-term impacts on mortality and CVD risks, which may inform future public health policies on DTR-related susceptible population and regions.

## 1. Introduction

Cardiovascular disease (CVD), including conditions that threaten the functions of human heart or blood vessels, is the principal cause of premature deaths and one of the most serious chronic health issues worldwide. As indicated by the Global Burden of Disease study, CVD was responsible for approximately 18.6 million deaths in 2019, accounting for 32% of the whole global mortality [1]. The underlying causes of CVD are dependent on the specific type. For instance, high burden of stroke, coronary artery disease, and peripheral artery disease could be attributed to numerous individual governed risk factors, e.g., unhealthy diet, physical inactivity, tobacco use, and alcohol consumption [2]. Apart from them, the impacts of exposures to environmental risk factors are also significant for understanding the burden of CVD.

Diurnal temperature range (DTR), defined as daily maximum minus minimum temperature, is widely used to reflect the within-day temperature variability [3,4]. It is an important meteorological indicator depicting the synchronism of ambient temperature. The deleterious short-term health effects of DTR exposure on daily timescales have long been established, as numerous studies provide growing epidemiological evidence on the positive associations of short-term DTR exposure with mortality/morbidity via time-series regression [5,6,7]. Nevertheless, the long-term health effects of chronic DTR exposure through population-based prospective cohort data have not been quantified thoroughly, especially on a national scale. The cohort study, different from the time-series study, could assess the cross-sectional long-term impact of environmental exposure on human health. Thus, it would be valuable especially for chronic medical conditions such as CVD. In past decades, the CVD-related mortality risk in low- and middle-income countries has rapidly increased, and contributed to at least 75% of the global CVD-related deaths [8]. Accounting for the CVD-induced substantial decrease in life quality and expectancy, and the heavy strain exerted on local health systems, quantitative estimation of CVD determinants such as DTR would be imperative to effectively prevent and manage CVDs, especially in densely populated low- and middle-income countries such as China [9,10].

The primary purpose of the present study was to investigate the longitudinal associations between DTR and all-cause mortality and cardiovascular incidences, in an effort to provide insights into the long-term health effects of DTR exposure. Moreover, this study sought to explore whether any subject characteristic could modify the effect strengths of the longitudinal relationships. This would be beneficial for people in the susceptible communities to develop effective plans on early prevention, recovery, and resilience of DTR-related CVD. Moreover, we can also project the DTR changes between the current and future climates across China under two representative climate change scenarios via the latest downscaled global climate models.

## 2. Methods

### 2.1. Study Population and Follow-Up

**Figure 1:**
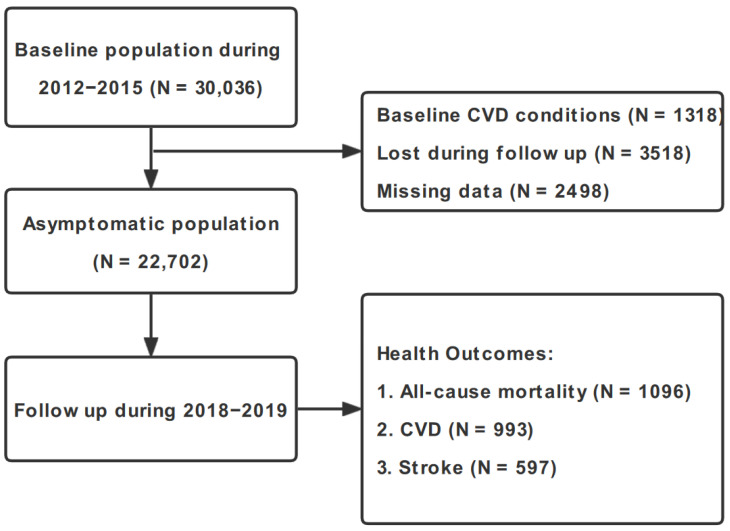
Flow diagram of inclusion and exclusion of CHS cohort participants. Abbreviations: CVD, cardiovascular disease; CHS, China Hypertension Survey.

We extracted the baseline study population from a nationwide representative large-scale cross-sectional study, namely the China Hypertension Survey (CHS), and obtained 30,036 participants (≥35 years) across 14 provinces in China during 2012–2015. The detailed descriptions concerning the CHS study design and population have been documented in our prior studies [11,12]. We conceived a follow-up survey on the CHS participants’ three health outcomes during 2018–2019, namely all-cause mortality, fatal and nonfatal CVD, and fatal and nonfatal stroke [13]. Inclusion and exclusion rules of participant selection in this study are outlined in Figure 1. The recruited subjects who were lost to follow-up (N = 3518), those who suffered from baseline CVD conditions (N = 1318), and those who had missing information on important risk factors at baseline year (N = 2498) were excluded. The overall follow-up rate was 84.5%, and no significant differences were detected in the baseline characteristics between subjects who were included in this study and those excluded due to the abovementioned reasons. Written informed consent was obtained from each study entrant. This study received the approval of the Ethics Committee of Fuwai Hospital (Beijing, China; approval no. 2018-1062, date of review: 17 July 2018).

### 2.2. Outcome Ascertainment

In this paper, we adopted all-cause mortality, fatal and nonfatal CVD, and fatal and nonfatal stroke as the main endpoints of interest. They were coded by local, trained medical workers based on the International Classification of Diseases, 10th Revision, Clinical Modification codes [14]. The hospital records or death certificates were further reviewed by the endpoint assessment committee of Fuwai Hospital to adjudicate the final diagnosis. In this study, all-cause mortality referred to death due to any cause. CVD consisted of stroke (I60–I61 and I63–I64), coronary heart disease (I20–I25), chronic heart failure (I50), and deaths owing to CVD (I00–I25, I27–I88, and I95–99). Stroke included fatal (i.e., subarachnoid hemorrhage, ischemic stroke, intracerebral hemorrhage, and unspecified stroke) and nonfatal stroke. Note that the fatal and nonfatal incident CVD outcomes were combined into the same group, lending elevated statistical confidence to the results [15,16].

### 2.3. DTR Exposure Assessment

We obtained the observed daily maximum, minimum, and mean near-surface air temperature (°C) and relative humidity (%) at ~2419 ground meteorological observations across China (Figure S1), with rigorous quality control from the China Meteorological Administration. The non-climatic biases brought by ground station relocations and observation system upgrades were corrected via multiple analyses of series for homogenization approach [17]. The long-term DTR exposure was defined as the baseline annual mean difference between daily maximum and minimum temperature. We first interpolated the daily meteorological station datasets into 1 km × 1 km grids by the Cressman interpolation method. Then, we assessed the address-based environmental exposures of each subject by averaging four grid points’ values within its boundary. To provide the overall picture of how the actual temperature is in China, we further explored the climate distributions in the main seven geographical areas of China (Figure S2), namely northeastern China, northern China, central China, eastern China, southern China, western China, and the Tibetan Plateau. The area-weighted averaged (i.e., explicitly use the area of each grid cell) observations were calculated to obtain the regional mean temperature and relative humidity.

### 2.4. Future Projection

To project future DTR changes in China, we used 31 global climate models archived in the latest “NASA Earth Exchange-Global Daily Downscaled Projections-Coupled Model Intercomparison Project Phase 6” (NEX-GDDP-CMIP6) [18]. Details about the 31 NEX-GDDP-CMIP6 models are shown in Table S1. The statistical downscaled projections of daily maximum and minimum temperatures in China during 2015–2100, with a horizontal resolution of 25 km × 25 km, were conducted via the Bias Correction Spatial Disaggregation method. Information about this downscaling method is described elsewhere [19]. Two Shared Socioeconomic Pathways (SSP) scenarios were selected, namely SSP2-45 and SSP5-85, outlining medium vs. non-mitigation of moderate vs. intense future warming, with the radiative forcing of 4.5 vs. 8.5 $W/m^{2}$, respectively, by the year 2100. We calculated the muti-model ensemble mean of difference of the projected daily maximum and minimum temperatures to evaluate the future DTR in China. A 10-year period centered on 2022 (i.e., 2016–2025) was selected to represent the present-day climate, while the period for 2051–2060 (2091–2100) was chosen for mid-term (long-term) future projection. The projection results were generally robust when we selected 20-year or 30-year periods. In general, the bias-corrected data showed higher skills in DTR projections at local to regional scales in China in comparison to their original CMIP6 counterparts. It would provide an added value on in-depth knowledge of potential future climate change patterns in China.

### 2.5. Covariate Measurement

The extensive baseline information of each participant was collected via the standardized questionnaires in face-to-face interviews, mainly including their demographic characteristics (age, gender, urban/rural residence, ethnicity, geographic region, and education level) and clinical characteristics (smoking status, alcohol consumption, body mass index [BMI], hypertension, hypercholesterolemia, diabetes mellitus, family history of CVD, and CVD medication history). The ethnicity was dichotomously categorized as Han and ethnic minorities. The geographic regions were classified into East, Central, and West China. Smoking status was defined as current, former, or never smokers. Current smokers are participants who have smoked ≥20 packets of cigarettes during their lifetime and remain smoking cigarettes currently; former smokers are those who have smoked ≥ 20 packets of cigarettes during their lifetime but quit smoking for ≥1 month; never smokers are those who never smoked or smoked <20 packets of cigarettes in their whole lifetime. Alcohol consumption referred to consuming ≥1 alcoholic beverage per week within the past month of the survey.

We calculated BMI via dividing weight by squared height ($\mathrm{kg}/m^{2}$), and classified it into underweight (≤18 $\mathrm{kg}/m^{2}$), normal (18−24 $\mathrm{kg}/m^{2}$), and overweight (≥24 $\mathrm{kg}/m^{2}$). Hypertension was defined as (1) the participant’s systolic blood pressure ≥140 mm Hg, and/or (2) diastolic blood pressure ≥90 mm Hg, and/or (3) having used antihypertensive medicine in the past two weeks. Hypercholesterolemia was defined as (1) total triglycerides ≥2.26 mmol/L, and/or (2) total cholesterol ≥6.22 mmol/L, and/or (3) high-density lipoprotein cholesterol <1.04 mmol/L, and/or (4) low-density lipoprotein cholesterol ≥4.14 mmol/L, and/or (5) using hypolipidemic medicine, and/or (6) prior diagnosis of dyslipidemia. Diabetes mellitus was defined as (1) fasting blood glucose ≥7 mmol/dL, and/or (2) prior diagnosis of diabetes, and/or (3) having used hypoglycemic medicine in the past two weeks. CVD medication history included angiotensin-converting enzyme inhibitors, angiotensin II receptor blockers, β-blockers, α-β-blockers, calcium channel blockers, centrally acting drugs, diuretics, vasodilators, as well as traditional Chinese medicine. The residential mobility information of each subject was surveyed as well during follow-up by asking, “Have you changed home address during follow-up period”.

We obtained monthly particles with an aerodynamic diameter of ≤2.5 μm (PM_2.5_) and nitrogen dioxide (NO_2_) concentrations of 15 km × 15 km horizontal resolution across China from the assimilation products of ~1000 surface air quality monitoring stations from the China National Environmental Monitoring Centre [20,21]. Their cross-validation with independent observations showed generally good $R^{2}$ values of 0.74–0.86. We derived the Normalized Difference Vegetation Index (NDVI), a surrogate of greenness, from the moderate resolution imaging spectroradiometer products (MOD13A3, 1 km × 1 km, monthly). The air pollutants and residential greenness exposure were assigned to each subject, similar to DTR. We further obtained annual average years of education and per capita gross domestic product (GDP) across survey counties in China from several sources (e.g., China Statistical Yearbook) as indicators of socioeconomic statuses.

### 2.6. Statistical Analysis

We depicted continuous and categorical variables via mean ± standard deviation and numbers (percentages), respectively. The ANOVA ($\chi^{2}$) test was employed in the comparison of continuous (categorical) variables among different groups. Multivariate Cox proportional hazards regression models were established to investigate the associations between long-term exposure to DTR and all-cause mortality and incident CVD outcomes. In light of previous DTR−CVD evidence, we built four models step by step with increasing degrees of underlying covariate adjustment. Specifically, Model 1 included age, sex, urbanity, ethnicity, geographic region, educational level, smoking status, alcohol consumption, and BMI. Model 2 added to Model 1 hypertension, hypercholesterolemia, diabetes mellitus, family history of CVD, and CVD medication history. Model 3 added to Model 2 the outdoor PM_2.5_ and NO_2_ concentrations and ambient mean temperature. According to previous studies [22,23], the social status and income were closely linked with the mortality and CVD risks as well. Therefore, Model 4 (i.e., fully adjusted multivariable model) further added county-level average years of education and per capita GDP. The hazard ratios (HRs) with corresponding 95% confidence intervals (95% CIs) were calculated for Models 1−4. We further conducted collinearity tests on the regression models, and the established models were reliable. We also calculated the incidence rates (per 1000 person-years) of three health outcomes via dividing incident cases by the total person-years of follow-up.

The concentration–response associations between DTR and cause-specific HRs were depicted using the restricted cubic spline model. We determined the degree of freedom according to the lowest Akaike information criterion value. Likelihood ratios were further calculated to examine the nonlinear associations through comparing the linear model fitness with that of the restricted cubic spline model. Given the potential nonlinearity, DTR was classified by tertiles as follows: ≤8.69 °C (lowest tertile), 8.69−11.11 °C (middle tertile), and ≥ 11.11 °C (highest tertile). The lowest tertile was regarded as the reference group. The HRs were presented for both continuous DTR and DTR tertiles. We evaluated the *p_trend_* by comparing the highest and the middle tertiles with the lowest (reference) tertile. The median values of each tertile were added into the Cox regression models on a continuous scale to obtain the *p_trend_*.

Several sensitivity analyses were performed to validate the robustness of our main conclusions. First, we used baseline DTR exposure of each month to examine the impact of seasonality. The warm (June to September), cold (December to March of the following year), and transitional season (April, May, October, and November) DTR exposure were employed as well [24]. Second, we excluded the subjects in the CHS cohort who died or developed disease within the first year after the baseline survey. Third, mean DTR exposure during the whole follow-up period was chosen to test the robustness of results on different exposure windows. Fourth, the subjects whose residential addresses were altered during follow-up were removed to evaluate the latent impacts of residential mobility. Fifth, NDVI was added additionally as a covariate into the regression models, as emerging evidence suggests a longitudinal association between residential greenness and CVD. Sixth, relative humidity was added as a covariate.

To identify susceptible subpopulations, we additionally implemented subgroup analyses by demographic (age, sex, and urbanity), clinical (alcohol consumption, and BMI), and environmental variables (PM_2.5_ and ambient mean temperature) and prior personal medical history (hypertension, hypercholesterolemia, and diabetes mellitus). Multiplicative cross-product interaction terms between DTR and the abovementioned variables were added to the fully adjusted Cox regression models to probe the potential effect modification.

The two-tailed p<0.05 was rendered statistically significant. All statistical analyses were performed with SAS version 9.4 (SAS Institute Inc, Cary, NC, USA).

## 3. Results

### 3.1. Baseline Characteristics of Study Participants

Table 1 presents the general baseline characteristics of CHS cohort participants overall and stratified by tertiles of DTR. Among the 22,702 eligible cohort members with the mean age of 56.1 (standard deviation: 13.1) years, we observed 1096, 993, and 597 incident cases of all-cause mortality, CVD, and stroke during the ~5-year follow-up survey, respectively (Figure 1). The cumulative incidence rates of all-cause mortality, CVD, and stroke were 10.49, 9.45, and 5.64 per 1000 person-years, respectively. There were relatively more females (53.7%) than males (46.3%) and more rural residents (55.4%) than urban residents (44.6%) in our cohort, and the majority of them were Chinese of Han ethnicity (89.5%). With increasing DTR tertiles (from tertile 1 to tertile 3), participants were younger and had a higher prevalence of overweight, hypertension, hypercholesterolemia, diabetes mellitus, and family history of CVD. In addition, participants with higher DTR were more likely to be exposed in less green, less humid, and colder environments (all *p* < 0.001).

**Figure 2:**
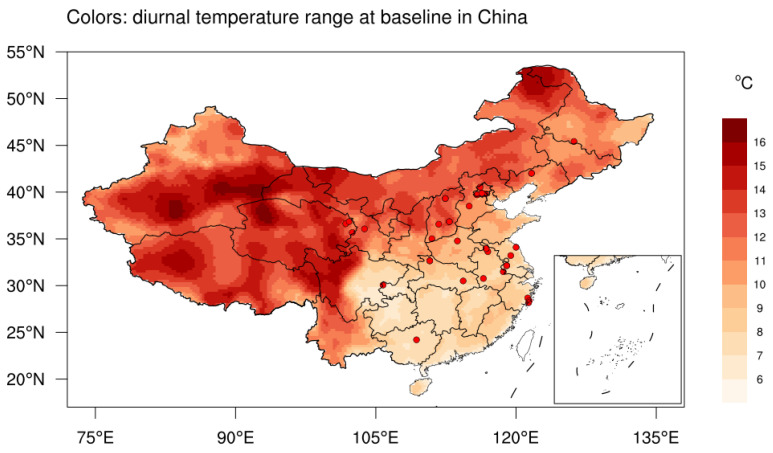
Baseline (2012–2015) annual mean DTR (colors, °C) and 30 China Hypertension Survey sites (red dots) in China (lack of data in Taiwan). Insets: South China Sea. Abbreviations: DTR, diurnal temperature range.

The baseline annual mean DTR had strong spatial heterogeneity, which featured higher values in northern vs. southern China, and in western vs. eastern China (Figure 2). Specifically, the eastern and southern China witnessed the minimum DTR in China (Table S2), with the regional mean values of 8.44 °C (maximum temperature 21.53 °C and minimum temperature 13.09 °C) and 8.87 °C (maximum temperature 22.78 °C and minimum temperature 13.91 °C), respectively. The maximum DTR was located in western China and Tibetan Plateau, with the regional mean values of 13.18 °C (maximum temperature 11.56 °C and minimum temperature −1.62 °C) and 13.33 °C (maximum temperature 6.54 °C and minimum temperature −6.79 °C), respectively. In addition, we observed an obvious declining trend of DTR over major parts of China for the past two decades, the magnitude of which was roughly −0.10 °C decade^−1^ (Figure S3).

### 3.2. Long-Term Impacts of DTR Exposure on Health Outcomes

Table 2 demonstrates the HRs and corresponding 95% CIs for the longitudinal associations between DTR exposure and three health outcomes in different models. Continuous analyses suggest a significantly positive relationship between long-term DTR exposure and all-cause mortality, with a 13% higher risk of all-cause mortality (HR = 1.13, 95% CI [1.08, 1.18], *p* < 0.001) based on a 1 °C increase in the DTR exposure in the fully adjusted multivariable model (i.e., Model 4). Likewise, subjects exposed to the highest tertile of DTR had a 70% higher risk of all-cause mortality (HR = 1.70, 95% CI [1.40, 2.07]) compared with those in the lowest tertile. The trend tests revealed a significant increasing trend of mortality risk with higher DTR tertile as well (*P_trend_* < 0.001).

We also detected the positive longitudinal associations between DTR exposure and CVD and stroke. In the fully adjusted multivariable model, the risk of developing CVD and stroke increased by 12% (HR = 1.12, 95% CI [1.07, 1.18], *p* < 0.001) and 9% (HR = 1.09, 95% CI [1.02, 1.16], *p* < 0.05), respectively, with DTR as the continuous variable. The HRs of developing CVD and stroke were 1.52 (95% CI [1.24, 1.87]) and 1.18 (95% CI [0.91, 1.53]) in the highest tertile, respectively, compared with their counterparts in the lowest tertile. The trend tests with higher DTR were significant for CVD (*P_trend_* < 0.001) and close to borderline significance for stroke (*P_trend_* = 0.071).

**Figure 3:**
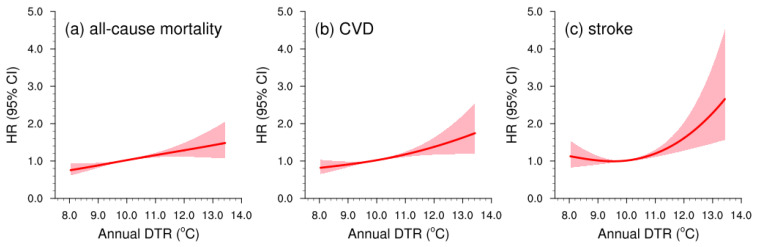
Concentration–response curves (red lines) with 95% CIs (shadings) of long-term DTR exposure with (**a**) all-cause mortality, (**b**) CVD, and (**c**) stroke. Abbreviations: DTR, diurnal temperature range; CVD, cardiovascular disease.

The concentration–response curves fitted by the restricted cubic spline model were linear for both all-cause mortality and CVD (*P_nonlinearity_* > 0.05; Figure 3). In addition, a U-shaped concentration–response curve was observed for stroke (*P_nonlinearity_* = 0.026), with a steeper slope at DTR exposure above 10 °C. We further conducted several sensitivity analyses to assess the robustness of the risk estimates. No statistically significant differences were observed in the longitudinal relationship between DTR exposure and three health outcomes by seasonality (each month of the baseline year or warm/cold/transitional season), excluding subjects, different exposure windows, or residential mobility (Table S3). Additional adjustment for baseline NDVI or relative humidity did not alter the main conclusions remarkably.

### 3.3. Subgroup Analysis and Effect Modification

**Figure 4:**
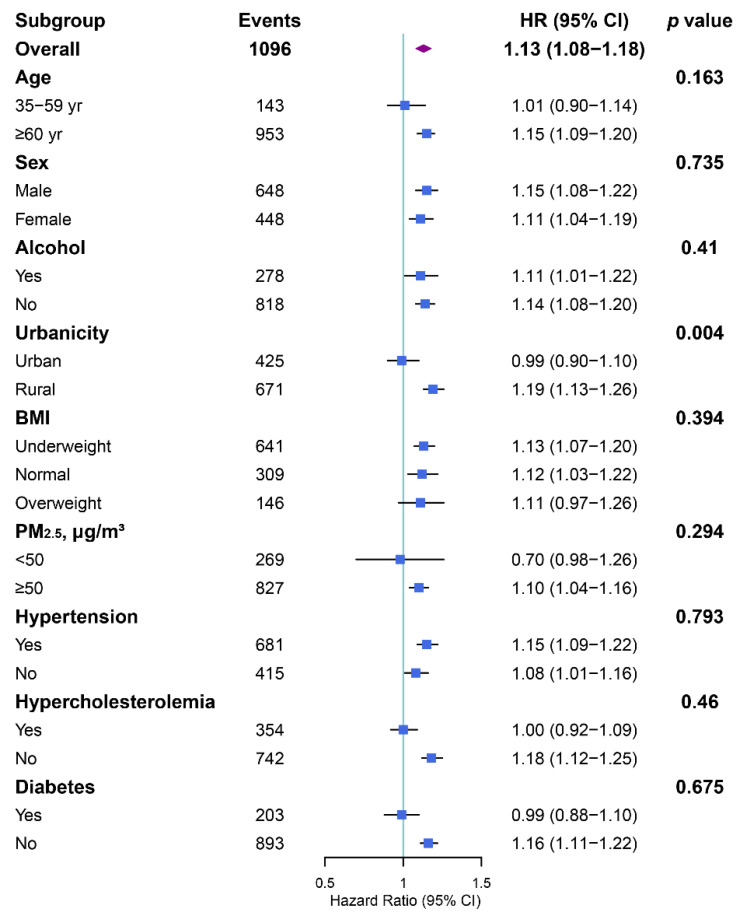
Subgroup analyses of hazard ratios (with 95% CIs) of all-cause mortality associated with a 1 °C increase in annual average DTR exposure. Abbreviations: DTR, diurnal temperature range; BMI, body mass index; PM_2.5_, particles with an aerodynamic diameter of ≤2.5 μm.

In the subgroup analyses, the effect of a 1 °C increment in DTR exposure on the risk of mortality or incident CVD outcomes did not differ by age, sex, alcohol consumption, BMI, PM_2.5_, and prior personal medical history (Figure 4, Figures S4 and S5). Nevertheless, the all-cause mortality risks were significantly higher among rural residents (HR = 1.19, 95% CI [1.13, 1.26]) than their urban counterparts (HR = 0.99, 95% CI [0.90, 1.10]) (*P_interaction_* < 0.01). As for stroke, we also found that the rural residents (HR = 1.12, 95% CI [1.03, 1.23]) might be more vulnerable to large DTR than urban residents (HR = 0.92, 95% CI [0.81, 1.05]) (*P_interaction_* < 0.01).

### 3.4. Future Projection of DTR in China

**Figure 5:**
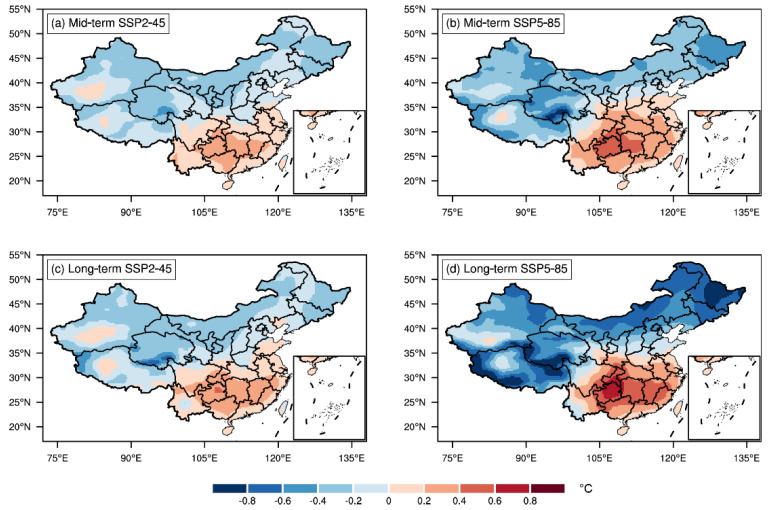
Spatial distribution of annual DTR changes for mid-term (2051–2060; (**a**,**b**)) and long-term (2091–2100; (**c**,**d**)) periods with respect to 2016–2025 in China under SSP2-45 (**a**,**c**) and SSP5-85 (**b**,**d**) scenarios.

Figure 5 displays the annual mean DTR changes in the mid-term and long-term SSP2-45 and SSP5-85 scenarios relative to the present-day climate. There is a remarkable dipole pattern in the future DTR changes, with an upward (downward) trend in southern (northern) China. The general spatial patterns bore notable similarities in the SSP2-45 and SSP5-85 projections. The magnitudes of DTR changes were, as expected, greater in the long-term than in the mid-term periods and in SSP5-85 than in SSP2-45 scenarios. To investigate the impact of seasonality, we further examined future DTR changes in the warm and cold season separately. We observed an increased DTR in the Tibetan Plateau during the warm season, which was opposite to the trend in the annual average (Figure S5). In addition, the future DTR changes in China were much more pronounced in cold seasons than in warm seasons (Figures S5 and S6).

## 4. Discussion

In this cohort study, we focused on the long-term effects of DTR on human health and firstly reported the positive longitudinal relationship between DTR exposure and three health outcomes in a population-based, nationwide, prospective cohort of adults aged 35 years and older. Our findings provide direct epidemiological evidence that the risks of all-cause mortality, CVD, and stroke elevated by 13%, 12%, and 9%, respectively, for each 1 °C increment in DTR after controlling for various confounding variables. Moreover, our estimated concentration–response curves were linear for all-cause mortality and CVD, and U-shaped for stroke. The U-shaped curves revealed that the stroke risks might increase faster as DTR reaches a relatively high level. The sensitivity analyses suggested that the DTR-related long-term health effects were generally robust regardless of seasonality, excluding subjects, different exposure windows, or adding additional covariates. Taking advantage of 31 state-of-the-art global climate models archived in the latest NEX-GDDP-CMIP6, we further projected the spatial variations of DTR in China under a warming climate. The bias-corrected high-resolution future projection indicated that the DTR would decrease (increase) in the southern (northern) China, with a greater magnitude in the cold season.

In the past, investigations of the deleterious health effects of DTR exposure have been almost exclusively concentrated on short-term exposures, i.e., days to weeks. For instance, Kan et al. [5] reported that a 1 °C increment in the 3-day running mean of DTR was responsible for a 1.37% increase in non-accidental mortality and a 1.86% increase in cardiovascular mortality. Yang et al. [6] suggested a 0.66% increase in stroke mortality per 1 °C increment in DTR at lag 0–10 days for 16 cities across China in 2007–2013. However, the present study indicates that there are chronic impacts of longer-term DTR exposure. The estimated long-term effects of DTR exposure in our study were greater than those of short-term effect studies, which was in line with the possibility of stronger, more persistent cumulative impacts from longer-term exposures. The results derived from this study are generally consistent with previous studies. Kang et al. [13] indicated a 6% increase in CVD per 1 °C increment in annual inter-day temperature variability in a representative cohort study in China. Zanobetti et al. [25] found that the long-term survival of elderly patients was significantly associated with summer temperature variability in four cohorts from 135 U.S. cities.

The stratified analyses could be instrumental in the early prevention of CVD based on diverse DTR susceptibilities. In the present study, rural residents were found to be more vulnerable than their urban counterparts to all-cause mortality and stroke. This could be partially explained by the fact that rural residents in China take on more agricultural labor in outdoor fields while urban residents are inclined to work in office buildings equipped with heating or air conditioning systems. Meanwhile, previous studies have indicated that the rural DTR is usually more violent than the neighboring urban one [26]. Hence, rural residents may generally be more sensitive to within-day temperature variability. Moreover, the higher all-cause mortality risks in rural residents can be attributed to the poor accessibility to hospitals in the rural areas of China in contrast to the quick and easy access to hospitals for urban residents.

The underlying pathways through which DTR acts upon mortality and CVD prevalence have not been well elucidated yet, but several plausible explanations exist. Emerging physiological evidence indicates a failure of the human thermoregulatory, nervous, and cardiovascular systems to adequately adjust to rapid within-day temperature variability [25]. Specifically, large DTRs might alter heart rate, arterial blood pressure, and inflammatory response, thereby enhancing the load on cardiovascular systems and further triggering CVD events, even deaths [27,28]. Moreover, large DTRs could also be unfavorable for outdoor physical exercise and healthy lifestyle, which may further harm cardiovascular functions [29]. In the present study, we also observed a slight increase with declining DTR in the HRs of the stroke group when the annual DTR was less than 10 °C (Figure 3c). One plausible mechanism of the DTR-related higher stroke risk is that the weak DTR (i.e., approaching daily maximum and minimum temperature) may disturb the physiological processes that regulate sleep and circadian rhythm [30]. For instance, when the daily maximum and minimum temperature are both high, it may be unfavorable for the nervous system to physically recover at night when it has already suffered from high temperatures in the daytime.

This study probed the present-day linear trends (Figure S2) and future changes (Figure 5) of DTR across China. The observed present-day DTR reduction in general parts of China may be associated with faster growing nocturnal minimum temperatures than diurnal maximum temperatures in the context of global warming during the past decades [31]. However, the conditions of the present day cannot infer the future, since the DTR exhibited significant declining trends over northern China and increasing trends over southern China in the future. This may be related to various processes such as cloud–climate feedback, land surface–air interaction, and planetary boundary layer processes [32]. For example, the enhanced tropospheric cloud cover can increase downwelling clear sky longwave radiation while decreasing downward solar radiation, leading to a reduced DTR. An interesting finding of the present study was that southern China currently with minimum DTR is expected to experience more violent intra-day temperature variability in the future, while northern China currently with relatively large DTR is projected to embrace declined DTR exposure in a warming climate. In this sense, the results in this study may also carry potential public health importance as they can inform the Chinese health authorities on future DTR-related decision programming and resource distribution. Moreover, the larger future DTR variations in cold seasons than in warm seasons implied that targeted measures may be taken in specific seasons to control the incidence of DTR-related CVD. In the future research, it would be preferable to assess the mortality and CVD burden attributable to long-term DTR exposure under different climate change and future population scenarios, and investigate the underlying drivers of corresponding patterns of changes.

Our present work has several strengths. To begin with, we first provided national epidemiological evidence on the long-term associations between DTR exposure and all-cause mortality and cardiovascular incidences in a nationwide cohort investigation. This establishment of the scientific basis would be informative for future DTR-related adaptation programs. In addition, the prospective design and representative general Chinese adults in our cohort increased the catholicity of the conclusions. Moreover, the temperature and humidity data from ~2419 ground meteorological stations as well as the daily downscaled projections from the latest NEX-GDDP-CMIP6 in China provided more accurate environmental exposure assessments, lending additional confidence to the results. Last, our analyses incorporated a relatively wide array of individual- and area-level potential confounders (including demographic, clinical, environmental, and socioeconomic ones) to adjust for the potential confounding.

Despite these advantages, several limitations of this study should be noted as well. First, limited by our data collection at baseline, the present study did not take into account the residual confounding brought by covariates such as dietary history, which may potentially bias the results. Therefore, caution should be taken when interpreting the overall association between DTR exposure and mortality or incident CVD outcomes shown in the results, which remain to be examined in future studies. Additionally, our cohort had a limited follow-up period of around 5 years. Subsequent analyses of a longer follow-up period would be preferable as our CHS cohort advances in the future.

## 5. Conclusions

In summary, based on a nationwide prospective cohort, we provided epidemiological evidence that chronic DTR exposure can exert long-term impacts on the risks of all-cause mortality and cardiovascular incidences after adjustment for a relatively rich variety of covariates. In addition, we projected a dipole pattern of future DTR changes across China by the end of the 21st century under global warming. Our results underscore the significance of strengthening the resilience of vulnerable regions with high DTR, and call for more public awareness of customized adaptation strategies to protect susceptible populations from detrimental health effects of DTR.

## Figures and Tables

**Table 1:** Baseline characteristics of China Hypertension Survey participants overall and stratified by tertiles of diurnal temperature range.

Variables	Total (n = 22,702)	Tertiles of DTR (°C)			*p* Value
		8.04−8.69 (n = 7808)	8.69−11.11 (n = 7029)	11.11−13.43 (n = 7865)	
Age, years	56.1 ± 13.1	57.0 ± 13.3	55.9 ± 13.2	55.5 ± 12.8	<0.001
Male, n (%)	10,505 (46.3)	3612 (46.3)	3379 (48.1)	3514 (44.7)	<0.001
Urban, n (%)	10,130 (44.6)	1780 (22.8)	4654 (66.2)	3696 (47.0)	<0.001
Han ethnicity, n (%)	20,315 (89.5)	7299 (93.5)	6980 (99.3)	6036 (76.7)	<0.001
Region, n (%)					<0.001
East	9263 (40.8)	5086 (65.1)	1433 (20.4)	2744 (34.9)	
Central	9460 (41.7)	1987 (25.4)	4909 (69.8)	2564 (32.6)	
West	3979 (17.5)	735 (9.4)	687 (9.8)	2557 (32.5)	
Educational to middle school or higher, n (%)	11,109 (48.9)	2638 (33.8)	4026 (57.3)	4445 (56.5)	<0.001
Smoking, n (%)					<0.001
Current	5731 (25.2)	2179 (27.9)	1686 (24.0)	1866 (23.7)	
Former	1216 (5.4)	392 (5.0)	359 (5.1)	465 (5.9)	
Never	15,755 (69.4)	5237 (67.1)	4984 (70.9)	5534 (70.4)	
Alcohol consumption, n (%)	6318 (27.8)	2096 (26.8)	2074 (29.5)	2148 (27.3)	<0.001
BMI (kg/m^2^), n (%)					<0.001
Underweight	10,213 (45.0)	4020 (51.5)	3062 (43.6)	3131 (39.8)	
Normal	8533 (37.6)	2733 (35.0)	2717 (38.7)	3083 (39.2)	
Overweight	3956 (17.4)	1055 (13.5)	1250 (17.8)	1651 (21.0)	
Hypertension, n (%)	8957 (39.5)	2997 (38.4)	2709 (38.5)	3251 (41.3)	<0.01
Hypercholesterolemia, n (%)	7724 (34.0)	2346 (30.0)	2514 (35.8)	2864 (36.4)	<0.001
Diabetes mellitus, n (%)	2286 (10.1)	714 (9.1)	661 (9.4)	911 (11.6)	<0.001
Family history of CVD, n (%)	2621 (11.5)	606 (7.8)	857 (12.2)	1158 (14.7)	<0.001
CVD medication history, n (%)	4929 (21.7)	1560 (20.0)	1364 (19.4)	2005 (25.5)	<0.001
PM_2.5_ (μg/m^3^)	61.7 ± 22.5	53.3 ± 11.2	76.7 ± 24.2	56.7 ± 22.9	<0.001
NO_2_ (μg/m^3^)	29.3 ± 13.3	26.7 ± 7.3	36.5 ± 14.9	24.8 ± 13.7	<0.001
NDVI	0.4 ± 0.1	0.6 ± 0.1	0.4 ± 0.1	0.3 ± 0.1	<0.001
Relative humidity (%)	66.1 ± 9.6	74.9 ± 2.7	66.2 ± 8.1	57.4 ± 7.1	<0.001
Ambient temperature (°C)	12.8 ± 4.8	16.9 ± 1.6	14.1 ± 2.9	7.6 ± 3.6	<0.001

Numbers are mean ± SD or no. (%). Abbreviations: DTR, diurnal temperature range; BMI, body mass index; CVD, cardiovascular disease; PM_2.5_, particles with an aerodynamic diameter of ≤2.5 μm; NDVI, normalized difference vegetation index.

**Table 2:** Hazard ratios and 95% CI for long-term diurnal temperature range with all-cause mortality, CVD, and stroke.

Outcomes	Per 1 °C Increment	Tertiles of DTR			*p*_trend_
		Lowest Tertile	Middle Tertile	Highest Tertile	
All-cause mortality					
No. of cases	1096	341	363	392	/
Incidence rate ^†^	10.49	10.29	10.01	11.18	/
Model 1 *^a^*	1.14 (1.09−1.18)	Reference	1.46 (1.23−1.74)	1.80 (1.52−2.14)	<0.001
Model 2 *^b^*	1.13 (1.09−1.18)	Reference	1.46 (1.23−1.74)	1.77 (1.49−2.10)	<0.001
Model 3 *^c^*	1.14 (1.10−1.19)	Reference	1.50 (1.25−1.79)	1.79 (1.51−2.13)	<0.001
Model 4 *^d^*	1.13 (1.08−1.18)	Reference	1.42 (1.17−1.74)	1.70 (1.40−2.07)	<0.001
CVD (fatal + nonfatal)					
No. of cases	993	285	328	380	/
Incidence rate ^†^	9.45	8.55	8.99	10.79	/
Model 1 *^a^*	1.13 (1.09−1.18)	Reference	1.04 (0.86−1.26)	1.68 (1.42−2.00)	<0.001
Model 2 *^b^*	1.13 (1.08−1.18)	Reference	1.06 (0.88−1.28)	1.63 (1.37−1.94)	<0.001
Model 3 *^c^*	1.14 (1.09−1.19)	Reference	1.09 (0.89−1.32)	1.66 (1.39−1.98)	<0.001
Model 4 *^d^*	1.12 (1.07−1.18)	Reference	0.94 (0.75−1.17)	1.52 (1.24−1.87)	<0.001
Stroke (fatal + nonfatal)					
No. of cases	597	191	192	214	/
Incidence rate ^†^	5.64	5.70	5.22	6.02	/
Model 1 *^a^*	1.11 (1.05−1.17)	Reference	0.82 (0.64−1.06)	1.45 (1.16−1.80)	<0.001
Model 2 *^b^*	1.10 (1.04−1.16)	Reference	0.84 (0.65−1.07)	1.40 (1.12−1.74)	0.003
Model 3 *^c^*	1.12 (1.06−1.19)	Reference	0.86 (0.67−1.12)	1.42 (1.14−1.77)	0.001
Model 4 *^d^*	1.09 (1.02−1.16)	Reference	0.68 (0.51−0.91)	1.18 (0.91−1.53)	0.071

† Incident rate per 1000 person years. ***^a^*** Model 1: adjusted for age, sex, urbanity, ethnicity, geographic region, educational level, smoking status, alcohol consumption, and BMI. ***^b^*** Model 2: Model 1 + hypertension, hypercholesterolemia, diabetes mellitus, family history of CVD, and CVD medication history. ***^c^*** Model 3: Model 2 + outdoor PM_2.5_ and NO_2_ concentrations, ambient temperature. ***^d^*** Model 4 (fully adjusted model): Model 3 + county-level average years of education and per capita GDP. Abbreviations: CVD, cardiovascular disease; DTR, diurnal temperature range; GDP, gross domestic product.

## Data Availability

All data that support the findings of this study could be available from the corresponding author upon reasonable request. Data is not publicly available due to privacy.

## Supplementary Materials

The following supporting information can be downloaded at: https://www.mdpi.com/article/10.3390/metabo12121287/s1, Figure S1: Geographical distributions of ~2419 meteorological stations in China (yellow dots), superimposed on the elevation (shading; m). Inset: South China Sea; Figure S2: The zoning map of seven geographical areas in China; Figure S3: Same as Figure 2, but colors for the linear trend of DTR (°C/decade) from 2000–2018 (divided by 10 years) in China. Inset: South China Sea; Figure S4: Same as Figure 4, but for CVD; Figure S5: Same as Figure 4, but for stroke; Figure S6: Same as Figure 5, but for warm season DTR changes; Figure S7: Same as Figure 5, but for cold season DTR changes; Table S1: Details of 31 global climate models employed in the present study for future projection; Table S2: The climatological (2000–2018) annual mean temperature and humidity characteristics in the seven geographical areas of China; Table S3: Sensitivity analyses for hazard ratios (95% CIs) of all-cause mortality, CVD, and stroke associated with a 1 °C increase in annual average DTR exposure; Text S1: List of the China Hypertension Survey Investigators.
