# Supplementary material for: Long-Term Impacts of Diurnal Temperature Range on Mortality and Cardiovascular Disease: A Nationwide Prospective Cohort Study
Source: Metabolites. 2022 Dec 19;12(12):1287. doi: 10.3390/metabo12121287 (PMC9784544; doi:10.3390/metabo12121287)
Supplement: Supplementary file 1 [file metabolites-12-01287-s001.zip › metabolites-1997034-supplementary.pdf]

Long-term impacts of diurnal temperature range on mortality and cardiovascular disease: A nationwide prospective cohort study

Haosu Tang[1,2,3], Xin Wang[4], Yuting Kang[5], Congyi Zheng[4], Xue Cao[4], Yixin Tian[4], Zhen Hu[4], Linfeng Zhang[4], Zuo Chen[4], Yuxin Song[4], Runqing Gu[4], Jiayin Cai[4], Gang Huang[1,2,3*], Zengwu Wang[4*], for the China hypertension survey investigators

[1]*State key Laboratory of Numerical Modeling for Atmospheric Sciences and Geophysical Fluid Dynamics (LASG), Institute of Atmospheric Physics, Chinese Academy of Sciences, Beijing, China*

[2]*Laboratory for Regional Oceanography and Numerical Modeling, Qingdao National Laboratory for Marine Science and Technology, Qingdao, China*

[3]*University of Chinese Academy of Sciences, Beijing, China*

[4]*Division of Prevention and Community Health, National Center for Cardiovascular Disease, National Clinical Research Center of Cardiovascular Disease, State Key Laboratory of Cardiovascular Disease, Fuwai Hospital, Peking Union Medical College & Chinese Academy of Medical Sciences, Beijing, China*

[5]*Office of the National Clinical Research Center for Geriatric Diseases, Beijing Hospital, National Center of Gerontology / Institute of Geriatric Medicine, Chinese Academy of Medical Sciences, Beijing, China*

Content

- Supplemental Figures S1–S7
- Supplemental Table S1–S3
- Supplemental Text S1

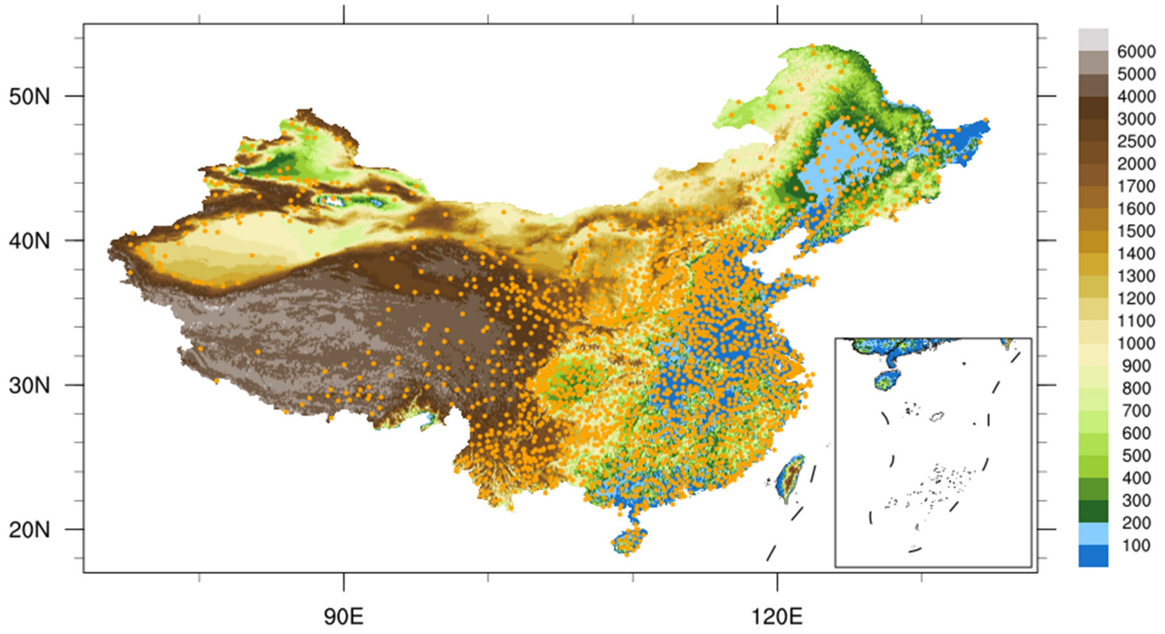

Figure S1. Geographical distributions of ~2419 meteorological stations in China (yellow dots), superimposed on the elevation (shading; m). Inset: South China Sea.

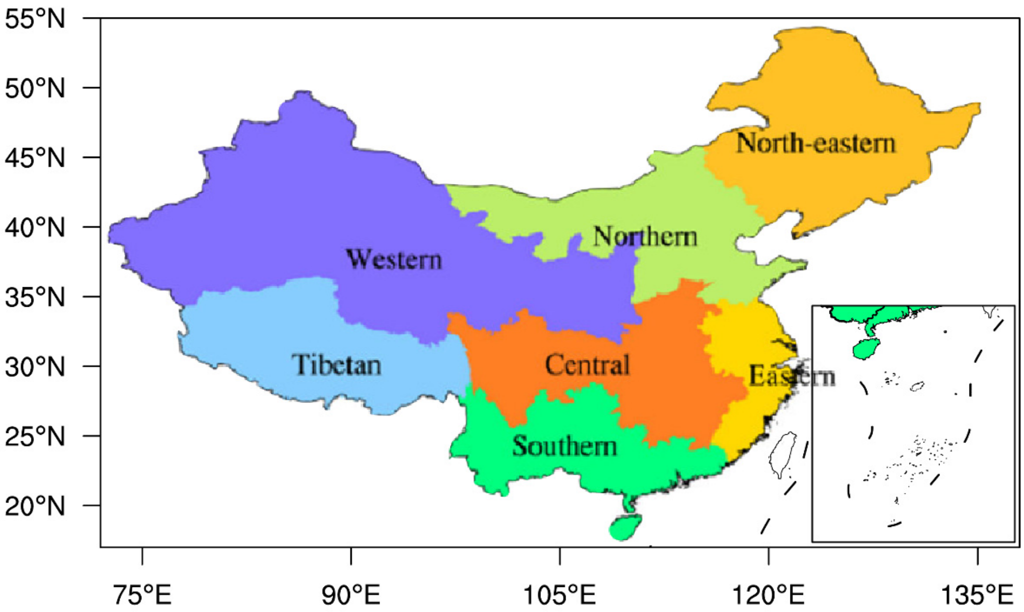

Figure S2. The zoning map of seven geographical areas in China.

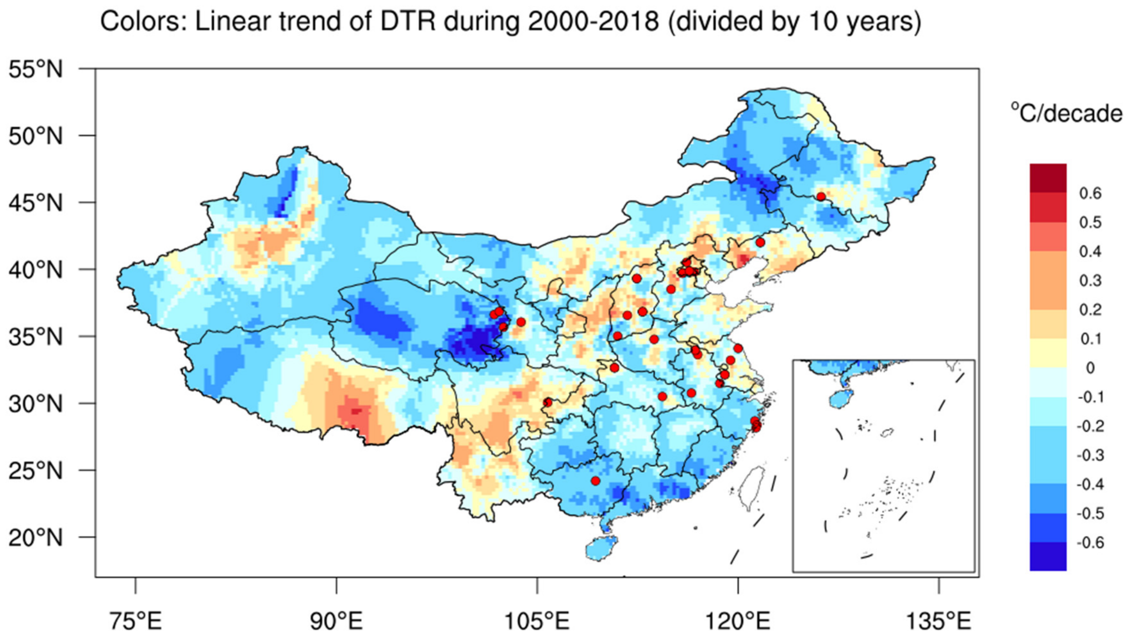

Figure S3. Same as Figure 2, but colors for the linear trend of DTR (°C/decade) from 2000–2018 (divided by 10 years) in China. Inset: South China Sea.

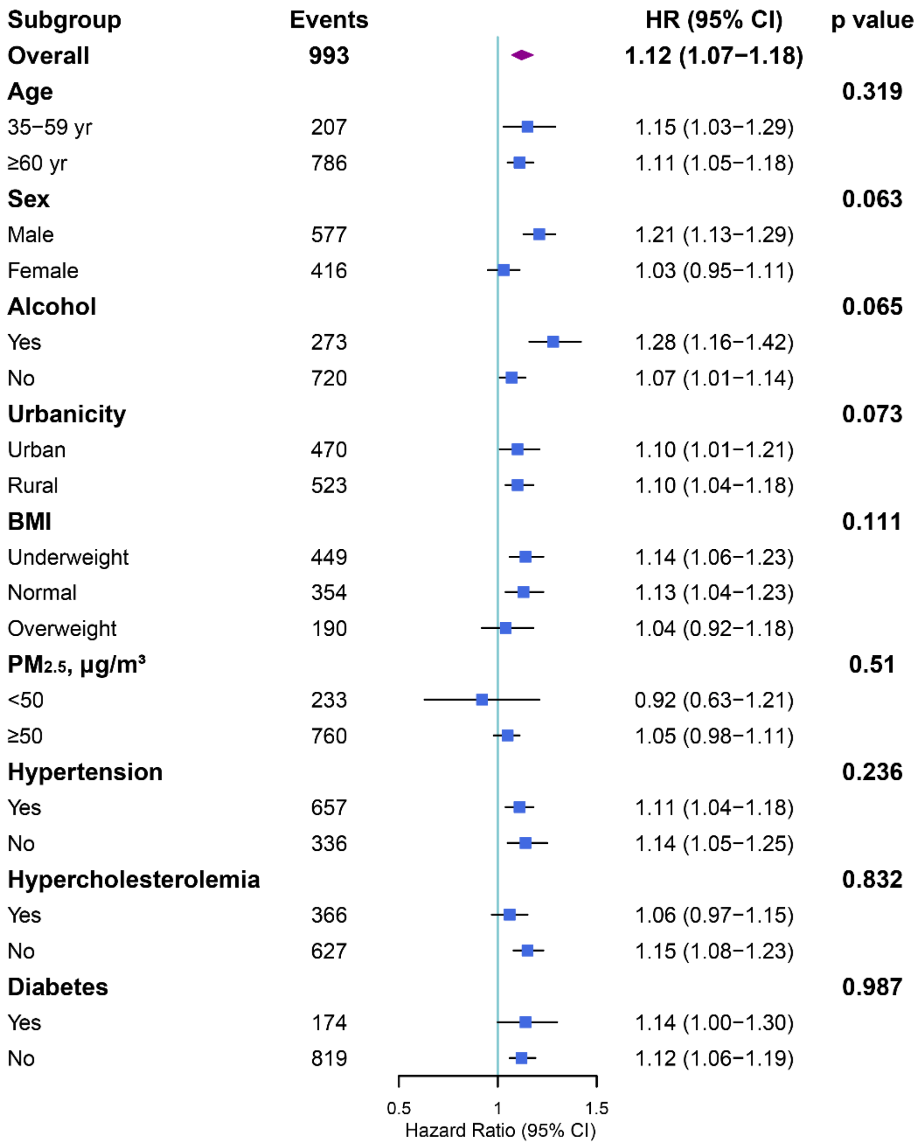

Figure S4. Same as Figure 4, but for CVD.

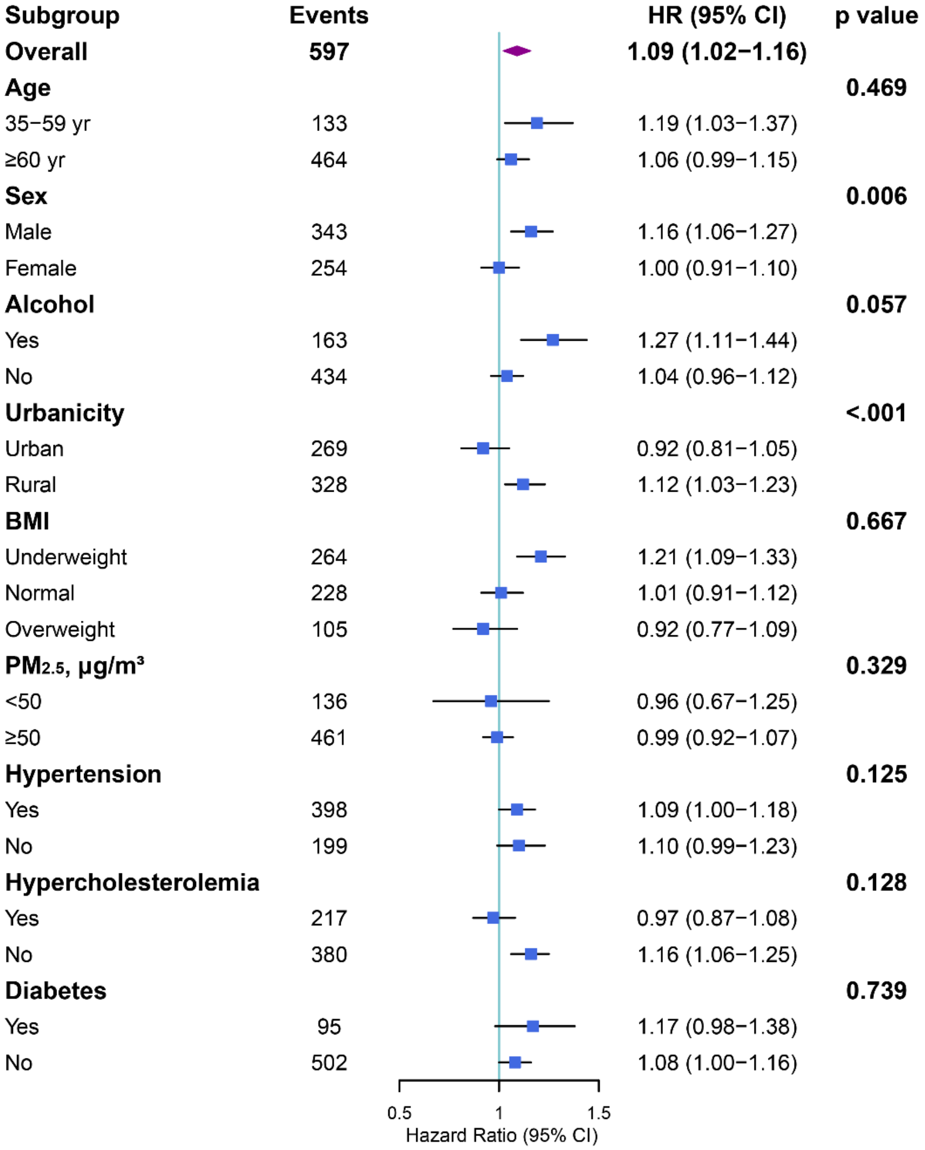

Figure S5. Same as Figure 4, but for stroke.

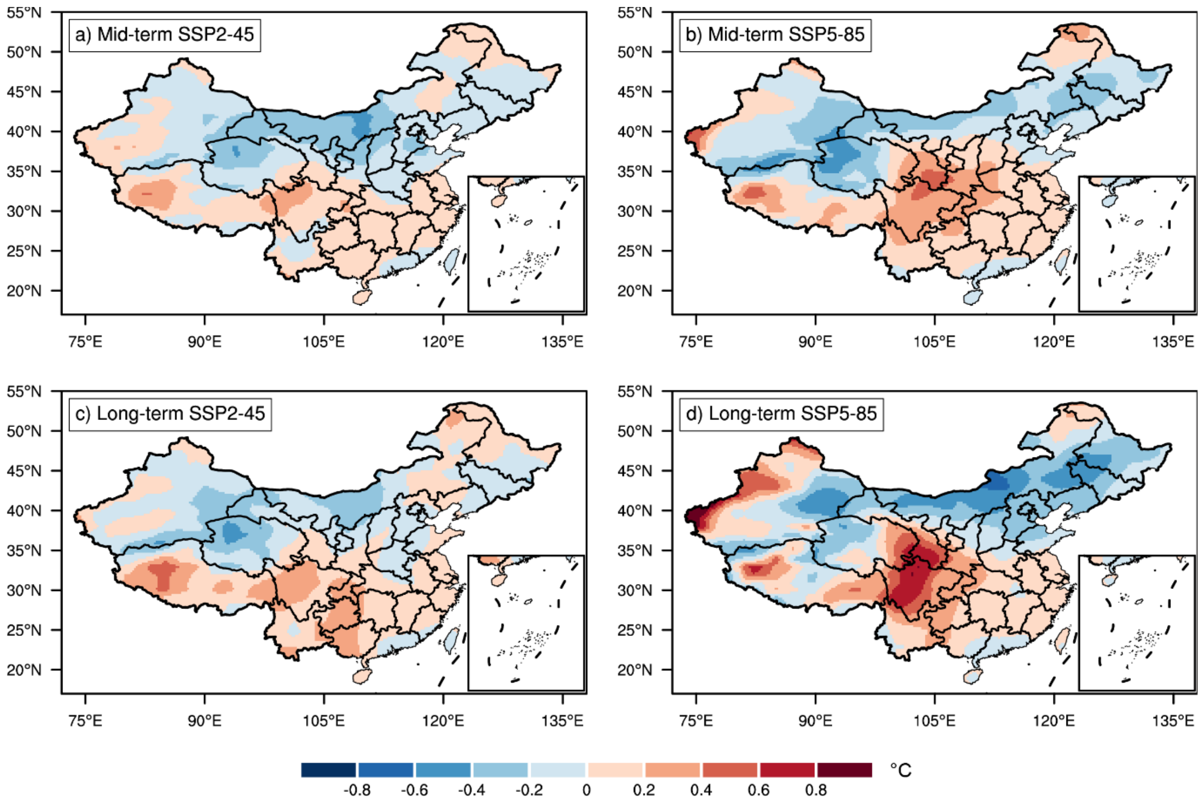

Figure S6. Same as Figure 5, but for warm season DTR changes.

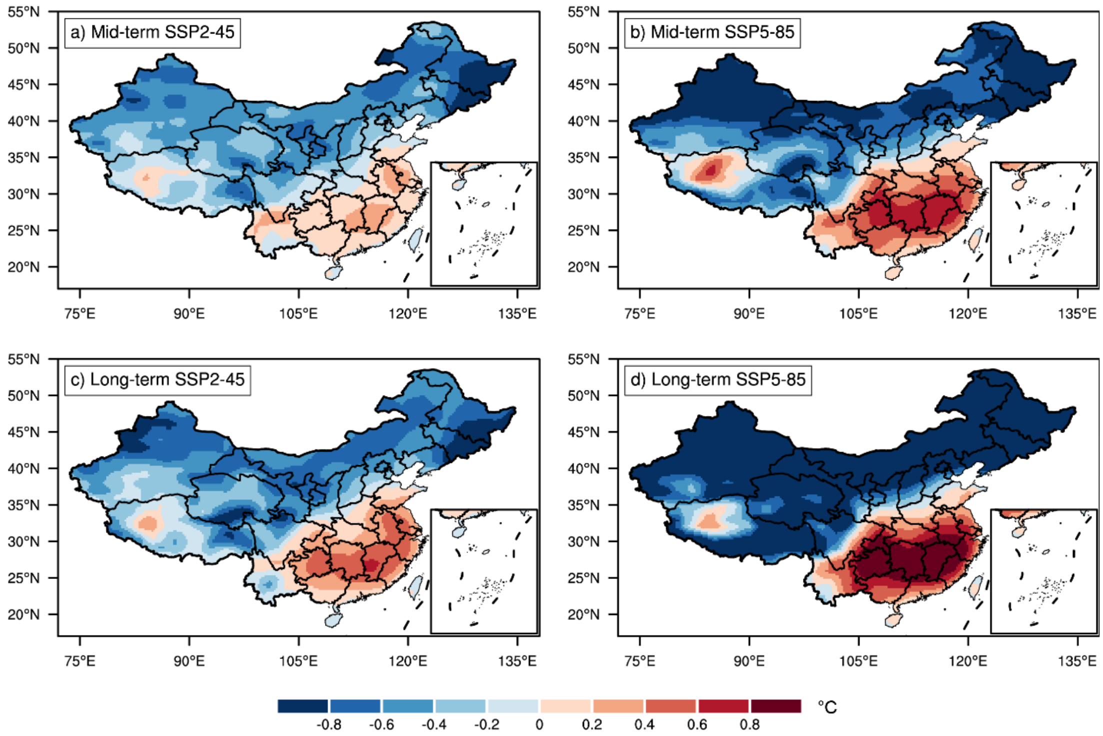

Figure S7. Same as Figure 5, but for cold season DTR changes.

Table S1. Details of 31 global climate models employed in the present study for future projection.

Model	Realization	Atmospheric Resolution		Institute/Region
		Latitude	Longitude	
ACCESS-CM2	r1i1p1f1	1.25°	1.875°	CSIRO–BOM/Australia
ACCESS-ESM1-5	r1i1p1f1	1.24°	1.875°	
BCC-CSM2-MR	r1i1p1f1	1.125°	1.125°	BCC-CMA/China
CMCC-CM2-SR5	r1i1p1f1	0.94°	1.25°	CMCC/Italy
CMCC-ESM2	r1i1p1f1	0.94°	1.25°	
CNRM-CM6-1	r1i1p1f2	1.4°	1.4°	CNRM-CERFACS/France
CNRM-ESM2-1	r1i1p1f2	1.4°	1.4°	
CanESM5	r1i1p1f1	2.81°	2.81°	CCCma/Canada
EC-Earth3	r1i1p1f1	0.7°	0.7°	EC-Earth-Consortium
EC-Earth3-Veg-LR	r1i1p1f1	1.125°	1.125°	
FGOALS-g3	r3i1p1f1	2.25°	2°	LASG-CESS/China
GFDL-CM4	r1i1p1f1	1°	1.25°	NOAA-GFDL/United States
GFDL-ESM4	r1i1p1f1	1°	1.25°	
GISS-E2-1-G	r1i1p1f2	2°	2.5°	NASA-GISS/United States
HadGEM3-GC31-LL	r1i1p1f3	1.25°	1.875°	MOHC/United Kingdom
HadGEM3-GC31-MM	r1i1p1f3	0.56°	0.83°	
INM-CM4-8	r1i1p1f1	1.5°	2°	INM/Russia
INM-CM5-0	r1i1p1f1	1.5°	2°	
IPSL-CM6A-LR	r1i1p1f1	1.26°	2.5°	IPSL/France
KACE-1-0-G	r1i1p1f1	1.25°	1.875°	NIMS-KMA/Korea
KIOST-ESM	r1i1p1f1	1.875°	1.875°	KIOST/South Korea
MIROC-ES2L	r1i1p1f2	2.81°	2.81°	MIROC/Japan
MIROC6	r1i1p1f1	1.4°	1.4°	
MPI-ESM1-2-HR	r1i1p1f1	0.94°	0.94°	MPI /Germany
MPI-ESM1-2-LR	r1i1p1f1	1.875°	1.875°	
MRI-ESM2-0	r1i1p1f1	1.125°	1.125°	MRI/Japan
NESM3	r1i1p1f1	1.875°	1.875°	NUIST/China

NorESM2-LM	r1i1p1f1	1.875°	2.5°	NCC-NMI/Norway
NorESM2-MM	r1i1p1f1	0.94°	1.25°	
TaiESM1	r1i1p1f1	0.94°	1.25°	RCEC-AS/China-Taiwan
UKESM1-0-LL	r1i1p1f2	1.25°	1.875°	MOHC/United Kingdom

Table S2. The climatological (2000–2018) annual mean temperature and humidity characteristics in the seven geographical areas of China.

Areas	DTR (°C)	maximum Ta (°C)	Minimum Ta (°C)	Relative humidity (%)
The whole China	11.68	13.66	1.99	57.77
Northeastern China	12.23	9.24	-2.99	62.97
Northern China	12.27	15.18	2.91	50.70
Central China	9.31	18.65	9.35	72.65
Eastern China	8.44	21.53	13.09	75.43
Southern China	8.87	22.78	13.91	76.44
Western China	13.18	11.56	-1.62	49.07
Tibetan Plateau	13.33	6.54	-6.79	41.58

Table S3. Sensitivity analyses for hazard ratios (95% CIs) of all-cause mortality, CVD, and stroke associated with a 1 °C increase in annual average DTR exposure.

Models	All-cause mortality	Fatal or nonfatal CVD	Fatal or nonfatal stroke
raw	1.13 (1.08−1.18)	1.12 (1.07−1.18)	1.09 (1.02−1.16)
1) January	1.09 (1.06−1.13)	1.13 (1.08−1.18)	1.15 (1.09−1.21)
2) February	1.06 (1.03−1.09)	1.07 (1.04−1.11)	1.04 (1.00−1.09)
3) March	1.08 (1.04−1.12)	1.08 (1.03−1.12)	1.05 (1.00−1.11)
4) April	1.06 (1.03−1.10)	1.06 (1.02−1.10)	1.04 (0.99−1.09)
5) May	1.14 (1.10−1.18)	1.12 (1.08−1.17)	1.10 (1.05−1.16)
6) June	1.11 (1.06−1.16)	1.11 (1.06−1.17)	1.06 (1.00−1.13)
7) July	1.11 (1.05−1.17)	1.11 (1.04−1.18)	1.10 (1.01−1.19)
8) August	1.06 (1.02−1.11)	1.04 (0.99−1.09)	0.97 (0.91−1.03)
9) September	1.09 (1.05−1.13)	1.07 (1.03−1.12)	1.04 (0.98−1.09)
10) October	1.14 (1.09−1.19)	1.14 (1.08−1.19)	1.11 (1.04−1.18)
11) November	1.11 (1.06−1.15)	1.06 (1.01−1.11)	1.05 (0.99−1.12)
12) December	1.16 (1.11−1.22)	1.12 (1.06−1.18)	1.13 (1.05−1.22)
13) Warm season	1.11 (1.06−1.17)	1.10 (1.04−1.16)	1.04 (0.97−1.12)
14) Cold season	1.12 (1.08−1.16)	1.11 (1.06−1.15)	1.09 (1.03−1.15)
15) Transitional season	1.13 (1.08−1.18)	1.12 (1.06−1.17)	1.09 (1.03−1.16)
16) Excluding subjects	1.13 (1.08−1.19)	1.12 (1.06−1.18)	1.08 (1.01−1.16)
17) DTR mean during follow-up	1.14 (1.09−1.19)	1.13 (1.08−1.19)	1.10 (1.03−1.17)
18) Residential mobility	1.11 (1.04−1.18)	1.10 (1.04−1.17)	1.08 (1.01−1.15)
19) Model 4 + NDVI	1.14 (1.08−1.20)	1.15 (1.09−1.22)	1.09 (1.01−1.18)
20) Model 4 + relative humidity	1.17 (1.11−1.24)	1.16 (1.09−1.23)	1.12 (1.03−1.22)

Abbreviations: CVD, cardiovascular disease; DTR, diurnal temperature range; NDVI, normalized difference vegetation index.

Text S1. List of the China Hypertension Survey Investigators.

This study could not be accomplished without the fine work of the staff at the national level. For a partial listing of colleagues see the follows (provinces sorted as alphabetical order):

Anhui: Liqun Hu, Hongqi Li, Qi Zhang, Guang Yan, Anhui Provincial Hospital, Hefei, Anhui, China; Fangfang Zhu, Anhui Institute of Cardiovascular Disease, Hefei, Anhui, China.

Beijing: Xianghua Fang, Chunxiu Wang, Shaochen Guan, Xiaoguang Wu, Hongjun Liu, Chengbei Hou, Xuanwu Hospital, Capital Medical University, Beijing, China.

Chongqing: Han Lei, Wei Huang, Nan Zhang, First Affiliated Hospital of Chongqing Medical University, Chongqing, China; Ge Li, Lihong Mu, Xiaojun Tang, Chongqing Medical University, Chongqing, China.

Fujian: Ying Han, Huajun Wang, Dongjie Lin Liangdi Xie, First Affiliated Hospital of Fujian Medical University, Fuzhou, Fujian, China; Daixi Lin, Fujian medical university, Fuzhou, Fujian, China.

Gansu: Jing Yu, Xiaowei Zhang, Wei Liang, Heng Yu, Qiongying Wang, Lanzhou University Second Hospital, Lanzhou, Gansu, China; Lan Yang, Maternal and Child Care Service Centre, Lanzhou, Gansu, China.

Guangdong: Yingqing Feng, Yuqing Huang, Guangdong General Hospital, Guangzhou, Guangdong, China; Peixi Wang, Jiaji Wang, Guangzhou Medical University, Guangzhou, Guangdong, China; Harry HX Wang, Sun Yat-Sen University, Guangzhou, Guangdong, China; Songtao Tang, Community Health Services Center of Liaobu, Dongguan, Guangdong, China.

Guangxi: Tangwei Liu, Rongjie Huang, Zhiyuan Jiang, Haichan Qin, First Affiliated Hospital of Guangxi Medical University, Nanning, Guangxi, China.

Guizhou: Guoqin Liu, Zhijun Liu, Wenbo Rao, Zhen Chen, Yalin Chu, Fang Wu, Zunyi Medical University, Zunyi, Gouzhou, China.

Hainan: Haitao Li, Jianlin Ma, Tao Chen, Hainan General Hospital, Haikou, Hainan, China; Ming Wu, Health and Family Planning Commission of Hainan, Haikou, Hainan, China.

Hebei: Jixin Sun, Yajing Cao, Yuhuan Liu, Center for Disease Prevention and Control of Hebei, Shijiazhuang, Hebei, China; Zhikun Zhang, Center for Disease Prevention and Control of Tangshan, Tangshan, Hebei, China; Yanmei Liu, Center for Disease Prevention and Control of Langfang, Langfang, Hebei, China;

Dejin Dong, Center for Disease Prevention and Control of Xingtai, Xingtai, Hebei, China; Guangrong Li, Center for Disease Prevention and Control of Dingzhou, Dingzhou, Hebei, China.

Heilongjiang: Hong Guo, Lihang Dong, Haiyu Zhang, Fengyu Sun, Xingbo Gu, Ye Tian, First Affiliated Hospital of Harbin Medical University, Haerbin, Heilongjiang, China.

Henan: Kaijuan Wang, Chunhua Song, Peng Wang, Hua Ye, Zhengzhou University, Zhengzhou, Henan, China; Wei Nie, Shuying Liang, Henan Academy of Medical Sciences, Zhengzhou, Henan, China.

Hubei: Congxin Huang, Fang Chen, Yan Zhang, Heng Zhou, Jing Xie, Jianfang Liu, Department of Cardiology, Renmin Hospital of Wuhan University, Wuhan, Hubei, China.

Hunan: Hong Yuan, Chengxian Guo, Third Xiangya Hospital, Central South University, Changsha, Hunan, China; Yuelong Huang, Biyun Chen, Center for Disease Control and Prevention of Hunan, Changsha, Hunan, China.

Inner Mongolia: Xingsheng Zhao, Wenshuai He, Xia Wen, Yanan Lu, Inner Mongolia people's hospital, Hohhot, Inner Mongolia, China.

Jiangsu: Xiangqing Kong, Ming Gui, Wenhua Xu, Yan Lu, Jun Huang, First Affiliated Hospital of Nanjing Medical University, Nanjing, Jiangsu, China; Min Pan, Affiliated Hospital of Nantong University, Nanjing, Jiangsu, China; Jinyi Zhou, Ming Wu, Center for Disease Control and Prevention of Jiangsu, Nanjing, Jiangsu, China.

Jiangxi: Xiaoshu Cheng, Huihui Bao, Xiao Huang, Kui Hong, Juxiang Li, Ping Li, Second Affiliated Hospital of Nanchang University, Nanchang, Jiangxi, China.

Jilin: Bin Liu, Junduo Wu, Longbo Li, Yunpeng Yu, Yihang Liu, Chao Qi, Second Hospital of Jilin University, Changchun, Jilin, China.

Liaoning: Jun Na, Li Liu, Yanxia Li, Guowei Pan, Center for Disease Prevention and Control of Liaoning, Shenyang, Liaoning, China; Degang Dong, Peng Qu, Health and Family Planning Commission of Liaoning, Shenyang, Liaoning, China.

Ningxia: Jinbao Ma, Health and Family Planning Commission of Ning Xia Hui Autonomous Region, Yinchuan, Ningxia, China; Juan Hui, Center for Disease Control and Prevention of Ning Xia Hui Autonomous

Region, Yinchuan, Ningxia, China; Fu Zhao, Health Supervision Institute of Xixia District in Yinchuan, Ning Xia Hui Autonomous Region, Yinchuan, Ningxia, China.

Qing Hai: Jianning Yue, Minru Zhou, Zhihua Xu, Xiaoping Li, Qiongyue Sha, Fuchang Ma, Qing Hai Center for Disease Control and Prevention, Xining, Qinghai, China; Qiuhong Chen, Huiping Bian, Qinghai Cardio-Cerebrovascular Disease Special Hospital, Xining, Qinghai, China.

Shaanxi: Jianjun Mu, Tongshuai Guo, Keyu Ren, Chao Chu, First Affiliated Hospital of Xi'an Jiaotong University, Xian, Shaanxi, China.

Shandong: Zhendong Liu, Hua Zhang, Yutao Diao, Shangwen Sun, Yingxin Zhao, Institute of Basic Medicine, Shandong Academy of Medical Sciences, Jinan, Shandong, China.

Shanghai: Junbo Ge, Jingmin Zhou, Xuejuan Jin, Jun Zhou, Zhongshan Hospital, Fudan University, Shanghai, China.

Shanxi: Bao Li, Lijun Zhu, Yuean Zhang, Gang Wang, Shanxi Cardiovascular Hospital, Taiyuan, Shanxi, China; Zhihan Hao, Wuxiang County People's Hospital, Wuxiang, Shanxi, China.

Sichuan: Li Cai, Zhou Liu, Zhengping Yong, Jianhong Tao, Yijia Tang, Sichuan Provincial People's Hospital, Chengdu, Sichuan, China; Shaoping Wan, Sichuan Cancer Hospital, Chengdu, Sichuan, China.

Tianjin: Zhenshan Jiao, Yuqiang Fan, Tianjin Academy of Traditional Chinese Medicine, Tianjin, China; Hui Gao, Wei Wang, Tianjin Municipal Commission of Health and Family Planning, Tianjin, China; Qingkui Li, Xiaomei Zhou, Tianjin Medical University, Tianjin, China.

Tibet: Yundai Chen, Bin Feng, Qinglei Zhu, Sansan Zhou, Chinese People's Liberation Army General Hospital, Lasha, Tibet, China.

Xinjiang: Nanfang Li, Lin Zhou, Delian Zhang, Jing Hong, People's Hospital of Xinjiang Uygur Autonomous Region, Urumuqi, Xinjiang, China.

Yunnan: Tao Guo, Min Zhang, First Affiliated Hospital of Kunming Medical University, Kunming, Yunnan, China; Yize Xiao, Center for Disease Prevention and Control of Yunnan, Kunming, Yunnan, China; Xuefeng Guang, Affiliated Yan'an Hospital of Kunming Medical University, Kunming, Yunnan, China.

Zhejiang: Xinhua Tang, Jing Yan, Xiaoling Xu, Li Yang, Aimin Jiang, Wei Yu, Zhejiang Hospital,

Hangzhou, Zhejiang, China.
